# Transit Amplifying Progenitors in the Cerebellum: Similarities to and Differences from Transit Amplifying Cells in Other Brain Regions and between Species

**DOI:** 10.3390/cells11040726

**Published:** 2022-02-18

**Authors:** Satoshi Miyashita, Mikio Hoshino

**Affiliations:** 1 Department of System Pathology for Neurological Disorders, Brain Research Institute, Niigata University, Niigata 951-8585, Japan; s.miyashita@bri.niigata-u.ac.jp; 2 Institute for Research Promotion, Niigata University, Niigata 951-8585, Japan; 3 Department of Biochemistry and Cellular Biology, National Institute of Neuroscience, NCNP, Tokyo 187-8502, Japan

**Keywords:** neural development, cerebellar granule cell prognitors, transit amplification, ATOH1, NEUROD1

## Abstract

Transit amplification of neural progenitors/precursors is widely used in the development of the central nervous system and for tissue homeostasis. In most cases, stem cells, which are relatively less proliferative, first differentiate into transit amplifying cells, which are more proliferative, losing their stemness. Subsequently, transit amplifying cells undergo a limited number of mitoses and differentiation to expand the progeny of differentiated cells. This step-by-step proliferation is considered an efficient system for increasing the number of differentiated cells while maintaining the stem cells. Recently, we reported that cerebellar granule cell progenitors also undergo transit amplification in mice. In this review, we summarize our and others’ recent findings and the prospective contribution of transit amplification to neural development and evolution, as well as the molecular mechanisms regulating transit amplification.

## 1. Introduction

How are complicated tissues created during tissue development? This is a central question in developmental biology. During development, a single fertilized egg becomes an individual through repeated cell division and differentiation. One of the most important steps in achieving these processes is the precise coordination of the number of cell divisions and the timing of differentiation. Transit amplification is one of the keys to such coordination and determination of the terminal cell number.

**Figure 1:**
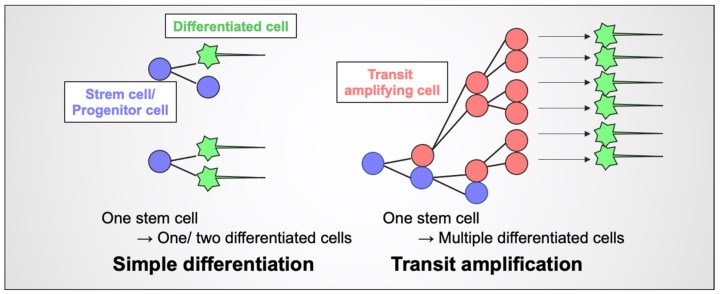
Schematic illustration of transit amplification. The simple differentiation model (**left**), such as direct neurogenesis of the developing cerebrum, is a process in which one stem cell generates one stem cell and one differentiated cell (**top**) or two differentiated cells (**bottom**). In the transit amplification model (**right**), one stem cell generates one stem cell and one transit amplifying cell; the latter undergoes a series of cell divisions to produce more differentiated cells.

During transit amplification, stem cells first divide to generate one stem cell and one transit amplifying cell; the latter subsequently undergoes a series of cell divisions (Figure 1) [1]. These “amplified” precursor cells are transient cells, which eventually become differentiated cells. This efficient system allows for (1) the production of large numbers of differentiated cells from a small number of original stem cells within a short period and (2) the reduction in the risk of DNA damage to or cell death of stem cells, which should be kept intact. Therefore, this system is widely adopted in various tissues (e.g., brain, intestine, and skin) [2,3,4]. However, the molecules and/or signaling pathways that regulate transit amplification during development, tissue homeostasis, and tissue regeneration remain to be elucidated.

The development of the central nervous system (CNS) provides an attractive model for this paradigm because of its well-studied developmental process, spatiotemporally distinct layers of stem cells and transit amplifying cells, and the well-established methods of plasmid transfer for the control of gene expression [5,6,7]. In this review, we first summarize the known transit amplification systems in the CNS. Thereafter, we will summarize the transit amplification system in the cerebellum [8,9]. Moreover, we will show that these transit amplifying cells are only present in mammals and contribute to the expansion of the mammalian cerebellum. As key molecules and signaling pathways expressed by the transit amplifying cells in the cerebellum are similar to those in the cerebral cortex, hippocampus, and lateral ventricle, elucidation of the molecular mechanisms that regulate the cerebellar transit amplification system may lead to a better understanding of the fundamental principles that regulate transit amplification in neural progenitors.

## 2. Traditional Models of Transit Amplification in Neural Progenitors

**Figure 2:**
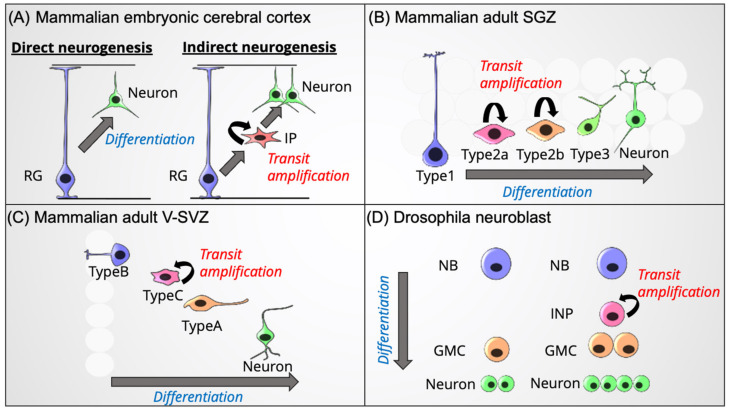
Schematic illustrations of transit amplification systems in the CNS. Transit amplification systems in the developing cerebral cortex (**A**), adult hippocampal SGZ (**B**), adult V-SVZ (**C**), and *Drosophila NBs* (**D**). Abbreviations: radial glia (RG), intermediate progenitor (IP), subgranular zone (SGZ), ventral subventricular zone (V-SVZ), neuroblast (NB), intermediate neural progenitor (INP), ganglion mother cell (GMC).

The size and complexity of the brain has evolved, giving rise to higher-order functions, such as cognitive ability, language, and sociability [10]. Primates’ brains, especially humans’, are larger and more complex than other mammals [11]. However, it is still unclear why mammals, especially humans, acquire such large brains. Transit amplification of neural progenitors (also called “indirect neurogenesis”) is an essential process in brain expansion [2]. Transit amplification has been observed in the cerebral cortex [6], hippocampus [12], and subventricular zone (SVZ) [13], suggesting that this system is a general principle of neuron generation in the amniote (Figure 2A–C). This system is also observed in *Drosophila* neuroblasts (NBs, Figure 2D), suggesting that it is highly conserved and utilized in the CNS of many species [14]. Interestingly, a recent study has revealed that mammals utilize this system more frequently than other animals, such as reptiles and avians, resulting in acquiring a larger neocortex [15].

In the remainder of this section, we summarize transit amplification/indirect neurogenesis in the CNS of mammals and Drosophila to explain (1) the differences and similarities between these traditional models (embryonic cerebellar cortex, adult subgranular zone [SGZ], and adult ventral SVZ[V-SVZ]) and a novel model in the cerebellum by describing the transit-amplification in the mammalian CNS, and (2) the evolutionary conservation of this transit-amplification system by describing the *Drosophila* neuroblast. Although transit amplification/adult neurogenesis has been reported in other species, such as birds [16] and fish [17], we will not assess these in this review.

### 2.1. Embryonic Neurogenesis in the Mammalian Cerebral Cortex

Transit amplification in the cerebral cortex (indirect neurogenesis) is well studied and characterized. In rodents, radial glial cells (RGs, a type of apical progenitor), which are in contact with the ventricular surface, proliferate symmetrically in the early embryonic period (~embryonic day [E]11) to expand the progenitor pool [6]. RGs gradually shift toward asymmetrical proliferation, generating one RG and one neuron around E12 (direct neurogenesis; Figure 2A left). This is followed by a period of indirect neurogenesis, with each RG generating one RG and one intermediate progenitor (IP, Figure 2A right). The IPs subsequently undergo one or a few mitoses, giving rise to postmitotic neurons and a rapid increase in the size of the cerebral cortex. Gene expression patterns during neurogenesis are well described [18]. In RGs, *Pax6* and *Sox2* are expressed first, followed by the increased expression of *Ngn1/2* and the differentiation of RGs into IPs. In the IPs, *Tbr2/Eomes* and *Neurod1* are expressed. After the termination of a series of cell divisions, postmitotic neurons express *Dcx*. Comprehensive molecular profiling of these progenitors has recently been performed with single-cell RNA sequencing (scRNAseq) [19,20,21].

In mammals that contain a gyrencephalic neocortex, there is another type of progenitor, namely, outer RGs (oRGs, also known as “basal progenitors” or “basal RG”). oRGs are located in the outer SVZ, just above the SVZ, and their processes extend only to the basal surface of the developing cortex [22,23,24]. The emergence of oRGs resulted in a more diverse pattern of neurogenesis: in the developing cortex in ferrets and humans, RGs asymmetrically generate one RG/one IP or one oRG/one RG. The oRG subsequently undergoes a series of cell divisions, followed by the generation of one oRG/one neuron, one oRG/one IP, or two oRGs [25]. Although oRGs have been observed in the developing cortex of ferrets, monkeys, and humans (and a very small population in mice), little is known about how these animals acquired this cell population. There is no doubt that these complicated patterns of neurogenesis contribute to the evolutionary expansion of the neocortex. This notion is further supported by a previous study in which neurogenesis was compared in multiple species, including mammals, reptiles, and birds [15]. Intriguingly, Cárdenas et al. [15] revealed that the expression ratio of *Robo1* and *Dll1* varies among species and determines the balance between direct and indirect neurogenesis. Recent studies have also been conducted to address other molecular mechanisms by which the balance between direct and indirect neurogenesis is regulated and the effect of the proliferation of transit amplifying cells on the number of neurons [26,27,28]. Importantly, during the development of the spinal cord, which has expanded less during evolution than the cerebral cortex and cerebellum, direct neurogenesis dominates in the generation of neurons from stem cells [15,29]. Taken together, the use of the transit amplification system (i.e., a shift from direct to indirect neurogenesis) is clearly related to the evolutionary expansion in the number of neurons and the size of the cerebral cortex. However, the identity of the molecules that regulate the emergence of indirect neurogenesis and diverse progenitors during cerebral cortical evolution remains elusive. In approaching these questions, we believe that it is important to use other model animals, particularly those with a gyrencephalic cortex and in which gene expression can be readily manipulated exogenously, such as ferrets.

### 2.2. Adult Neurogenesis in Mammals

In the adult brain, neurogenesis is also observed in specific regions, although its frequency is much less than that in the embryonic brain. Transit amplification of neural progenitors has been observed during adult neurogenesis in the subgranular zone (SGZ) of the hippocampal dentate gyrus and the ventral subventricular zone (V-SVZ) attached to the lateral ventricle (LV, Figure 2B,C). We briefly summarize transit amplification in both these regions, as well as adult neurogenesis via transit amplification in other brain regions.

#### 2.2.1. SGZ of the Hippocampal Dentate Gyrus

Adult neurogenesis in the SGZ of the hippocampal dentate gyrus was first discovered via 3H-thymidine labeling in the rat brain more than half a century ago [30]. Subsequently, adult hippocampal neurogenesis has been observed in other mammals, including humans, clearly indicating that it is an evolutionarily conserved process [31,32,33]. Adult neural stem cells (NSCs, also called Type-1 cells or radial glia-like cells), which are located in the SGZ, exhibit a horizontal or radial shape and maintain a quiescent state (Figure 2B) [11]. After self-renewal, they generate proliferative intermediate cells called Type-2 cells. Type-2 cells can be sub-divided into Type-2a and Type-2b cells based on their gene expression profiles. Type-2a cells express *Ascl1*, *Tbr2*/*Eomes*, *Ngn2*, *Sox1*/*2*, *Hes5*, *Gfap*, *Glt*, and *Nestin*, which are common features of NSCs. Type-2a cells subsequently turn into Type-2b cells with the expression of neurogenic genes, such as *Neurod1* and *Dcx*. Subsequently, Type-2b cells give rise to migratory Type-3 cells (also called NBs), which express *Prox1*, followed by termination of the cell cycle and maturation into neurons. Type-2 and Type-3 cells are responsible for transit amplification, and extrinsic stimuli are known to affect the production of newborn neurons (related to, e.g., voluntary movement, stress, aging, and epilepsy). However, further studies are needed to fully elucidate the molecular mechanisms that regulate transit amplification in the hippocampus.

#### 2.2.2. V-SVZ of the LV

Another transit amplifying progenitor is located in the V-SVZ, which is the source of newborn neurons in the olfactory bulb (OB) [13,34]. NSCs in the V-SVZ are called Type-B1 cells; they are in contact with the surface of the LV (Figure 2C). Type-B cells exhibit astrocyte-like gene expression profiles, expressing *Gfap*, *Slc1a3*/*Glast*, and *Fabp7*/*Blbp* [35]. Type-B cells are slowly dividing NSCs that generate Type-C cells. Type-C cells are transit amplifying progenitors in the V-SVZ and express *Ascl1*, *Pax6*, and *Neurogenin2* (*Ngn2*), which seemingly determine the neurogenic potential of Type-C cells. In the mouse V-SVZ, Type-C cells symmetrically divide one to three times and give rise to Type-A cells that subsequently migrate rostrally, over a long distance, to the OB, with repeated cell divisions [36]. In contrast, Wang et al. [37] reported that in the adult human brain, the proliferation of Type-A cells was barely observed. Interestingly, clonal analysis in the adult mouse brain revealed that Type-B and Type-C cells generate *Olig2*-positive oligodendrocyte progenitor cells, which differentiate into mature oligodendrocytes that express NG2 [38]. The production of oligodendrocytes is increased by demyelinating lesions [39]. These results suggest that Type-B cells and a small population of Type-C cells in the V-SVZ have the potential to generate oligodendrocytes as well as a variety of interneurons.

#### 2.2.3. Other Regions

In addition to the SGZ and the SVZ, further studies have revealed the hypothalamus, amygdala, and striatum as niches of adult neurogenesis [39,40,41,42,43,44]. In the hypothalamus, adult NSCs, called tanycytes, divide asymmetrically to produce one NSC and one neural, glial, or oligodendrocyte progenitor cell [41]. These progenitors may undergo a limited number of cell divisions and differentiate into neurons, astrocytes, or oligodendrocytes.

Ernst et al. first reported newborn neurons in the adult striatum of the human brain [44]. Newborn neurons have also been observed in the adult striatum of other mammals [45,46]. The source of these cells is still unclear, but the SVZ is one candidate. If so, a transit amplification system may be involved in the generation of striatal neurons, such as that of newborn neurons in the OB.

### 2.3. Neurogenesis in the Drosophila CNS

In addition to in the mammalian brain, transit amplification of neural progenitors is observed in the *Drosophila* CNS, suggesting that this system is well conserved during evolution [47,48,49]. During neurogenesis in *Drosophila* embryos and larvae, type-I NBs, regarded as stem cells in the *Drosophila* brain and originate from neuroepithelial cells, proliferate via asymmetric generation of one NB and one ganglion mother cell (GMC, Figure 2D). GMCs subsequently divide only once to give rise to neurons or glial cells in the embryonic CNS. In contrast, type-II NBs in the larval CNS, which are relatively recent to science, generate intermediate neural progenitors (INPs, also called transit amplifying GMCs). Thereafter, INPs undergo several cell divisions to generate one INP and one GMC; the latter generates two neurons. Therefore, transit amplifying systems in the type-II NB lineage can produce more neurons than those in the type-I NB lineage. Because of its relevance to indirect neurogenesis in the mammalian cortex or adult neurogenesis in the SGZ or SVZ, neurogenesis in *Drosophila* is a promising model for analyzing the principle of transit amplification during neural development.

## 3. Transit Amplification of Mammalian Cerebellar Granule Cell Progenitors

Cerebellar granule cells (GCs) are excitatory interneurons in the cerebellar cortex and are the most abundant neurons in the mammalian brain. In humans, GCs account for more than 80% of all neurons in the brain [50] (60% in mice [51]). This implies an association between GCs and the acquisition of higher-order cognitive functions, in addition to their conserved role in motor coordination and motor learning.

Cerebellar GCs originate from neural progenitors in the upper rhombic lip (uRL) of the embryonic cerebellar primordium [52,53]. During E10–E16, fate-determined neural progenitors in the uRL, which express the basic helix–loop–helix transcription factor *Atoh1*, generate glutamatergic neurons of the deep cerebellar nuclei (Glu-DCN neurons), unipolar brush cells (UBCs), and GC progenitors (GCPs). As GCPs continue to proliferate after leaving the uRL, GCPs are regarded as transit amplifying cells. On the other hand, Glu-DCN neurons and UBCs become postmitotic neurons directly after leaving the uRL. The GCPs subsequently migrate beneath the pial surface and form the external GC layer (EGL), the transient germinal zone for the GC lineage. GCPs explosively proliferate in the EGL from approximately E16.5 to 2–3 weeks after birth in the mouse cerebellum, resulting in a 1000-fold increase in cerebellar volume from the embryonic to the adult cerebellum [54].

**Figure 3:**
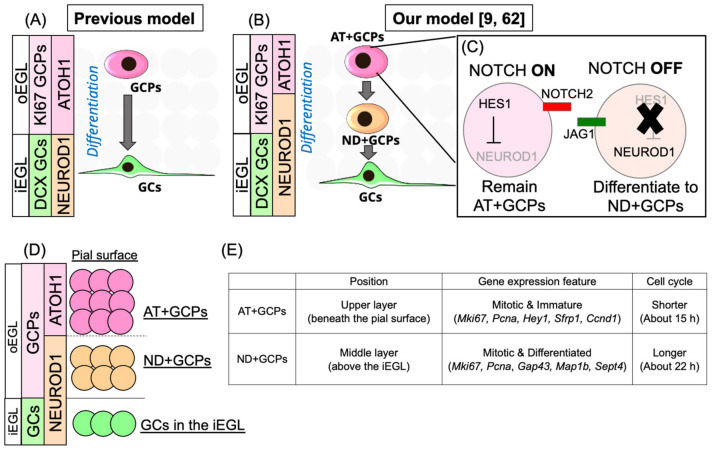
Novel developmental machinery of mammalian granule cells in the EGL. Schematic illustrations of the previous model (**A**) and our model (**B**, referencing our recent studies [9,62]) of GC development in the EGL. Notch-signaling activity and the regulation of the NEUROD1 protein in the AT + GCPs (**C**). Layer structure (**D**) and features of AT + GCPs and ND + GCPs (**E**). AT + GCPs are observed in the upper part of the EGL (beneath the pial surface) and ND + GCPs in the middle of the EGL (above the iEGL, which contains GCs). Both AT + GCPs and ND + GCPs are mitotic and express *Mki67* and *Pcna*. AT + GCPs express genes involved in maintaining the immature and undifferentiated state, such as *Hey1, Sfrp1, and Ccnd1*, while ND + GCPs express genes involved in the differentiation into neurons, such as *Gap43, Map1b, and Sept4.* The cell cycle length and G1 length of AT + GCPs are shorter than those of ND + GCPs. Abbreviations: granule cell progenitors (GCPs), granule cells (GCs), ATOH1 positive GCPs (AT + GCPs), NEUROD1 positive GCPs (ND + GCPs), external granule cell layer (EGL), inner EGL (iEGL), outer EGL (oEGL).

After GCPs leave the uRL, they continue to express *Atoh1* and start to express other transcription factors, such as *Pax6*, *Hes1*, and *Hey1*/*2*. In addition, *Sox2,* which is expressed by the stem cells in the cerebral cortex, SGZ, and V-SVZ, is also expressed in the EGL for a very limited period. Postmitotic GCs subsequently express *Dcx*, an immature neuronal marker, and migrate tangentially in the EGL and radially from the inner EGL (iEGL) into the internal GC layer (IGL, Figure 3A). Recent studies have revealed various molecules and signaling pathways that regulate the development of GCs. [55,56,57,58]. However, the identity of the molecular machinery that regulates the evolutionary expansion of the cerebellum and the mechanism by which expansion of the cerebellum contributes to its cognitive functions remain enigmatic.

### 3.1. Identification of Novel Transit Amplifying Progenitors in the Mouse EGL

In the classic model of cerebellar GC development described above, GCPs in the mammalian EGL are considered transit amplifying cells, uniformly expressing *Atoh1* and becoming postmitotic GCs directly after ATOH1 protein expression ceases (Figure 3A). However, our group and another have confirmed that there are ATOH1-negative populations located just beneath the ATOH1-positive cells in the EGL that, surprisingly, remain mitotic [59,60]. In a previous report, we also noted that the protein expression of NEUROD1, a transcription factor, was expressed in ATOH1-negative GCPs [9]. A recent study also confirmed these expression patterns in the mouse cerebellum [61]. We observed that most GCPs express either the ATOH1 or the NEUROD1 protein. Since ATOH1- and NEUROD1-expressing GCPs have distinct characteristics, in terms of their localization, gene expression, and cell cycle length, we named these two types of GCPs as “AT + GCPs” and “ND + GCPs”, respectively (Figure 3B,D,E). AT + GCPs are located more superficially (beneath the pial surface) in the outer EGL (oEGL), while ND + GCPs reside just beneath the AT + GCP layer and just above the iEGL, where postmitotic GCs reside (Figure 3D). Eventually, AT + GCPs and ND + GCPs constitute distinct layers within the oEGL. The cell cycle length of AT + GCPs is shorter than that of ND + GCPs (15 h vs. 21 h, Figure 3E). Differential gene expression analyses suggested that AT + GCPs are more proliferative and immature (characterized by the expression of *Hey1*, *Sfrp1*, *Ccnd1*, etc.), while ND + GCPs are less proliferative and more differentiated (characterized by the expression of *Gap43*, *Map1b*, *Sept4*, etc., Figure 3E). Furthermore, trajectory analysis of the single cell RNA-seq data of the postnatal cerebellum demonstrated a differentiation trajectory from AT + GCPs to ND + GCPs to GCs (unpublished data). In addition, a small number of GCPs located at the border between AT + GCPs and ND + GCPs express both ATOH1 and NEUROD1. These findings suggested that AT + GCPs give rise to ND + GCPs (called “AT-ND transition” [9]), which differentiate into GCs (Figure 3D), as well as the presence of a two-step amplification system of GCPs in the EGL.

As the diversity of neural progenitors in the developing cerebral cortex is associated with evolutionary cortical expansion, we believe that the diversity of GCPs, including the ancestral cells in the uRL, is also an important step in cerebellar expansion.

The diversity of GCPs, which is marked by the activity of Notch signaling, has also been reported recently [62]. Adachi et al. [62] proposed a model in which GCPs with high Notch-signaling activity continue to proliferate (ON-GCPs) by the HES1-mediated suppression of NEUROD1 protein expression; this suppresses the differentiation of GCPs into GCs [63]. In contrast, GCPs with low Notch-signaling activity (OFF-GCPs) start to differentiate. We observed salt-and-pepper (nonuniform) expression patterns of *Hes1* and *Notch2* only in AT + GCPs, while the expressions of these were consistently low in ND + GCPs (unpublished data). Taken together, these results suggest that Notch signaling, which is only activated in AT + GCPs, subdivides AT + GCPs into two subgroups (ON-AT + GCPs and OFF-AT + GCPs, Figure 3C). As Notch signaling oscillates in neural progenitors [64], it was assumed to oscillate in AT + GCPs as well. However, the dynamics of Notch-signaling activities in AT + GCPs remain elusive.

### 3.2. Comparisons of Transit Amplification of GC-Lineage Cells in Vertebrates

**Figure 4:**
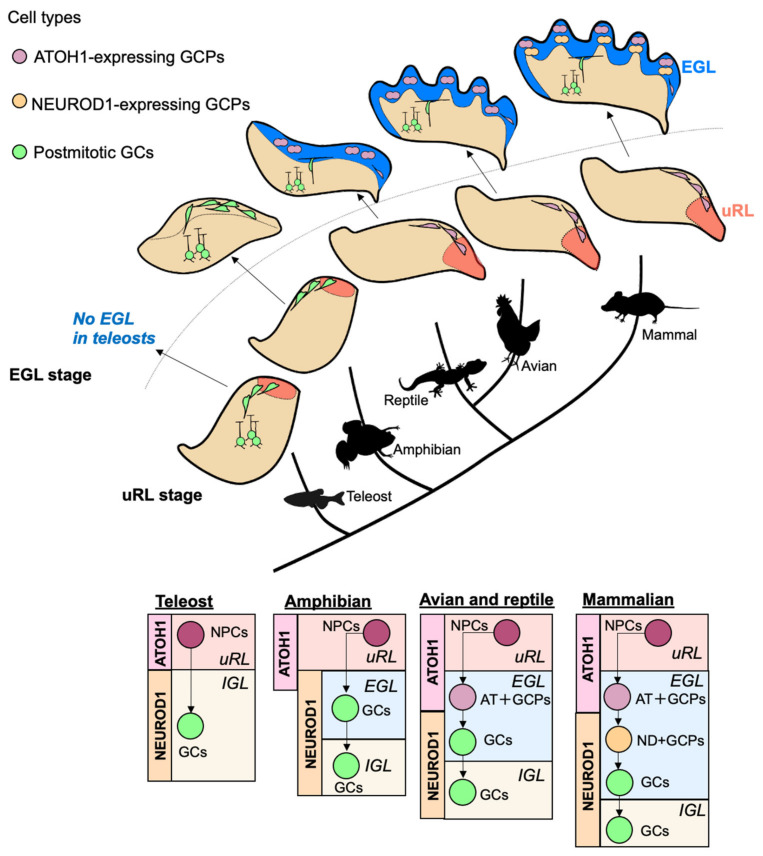
Dynamic changes in GC development in vertebrate evolution. In the teleost uRL (red area in the drawing), neural progenitor cells (NPCs) express atoh1 a/b, homologs of mammalian ATOH1, and directly generate GCs which migrate to the IGL without forming an EGL (in this figure, we labeled atoh1 a/b as “ATOH1” to emphasize the similarities and dissimilarities among species). In the amphibian, reptile, avian, and mammalian uRL, NPCs also express ATOH1. In these animals, however, after the NPCs are committed to becoming GCPs and the GCPs leave the uRL, the GCPs migrate along the pial surface of the cerebellum to form the EGL (blue area in the drawing). The GCPs then proliferate, differentiate in the EGL, and migrate to the IGL, terminating proliferation. Interestingly, GCPs are not proliferative and do not undergo cell divisions in the amphibian EGL, possibly because of the strong expression of the NEUROD1 protein in the outer part of the EGL, whereas the NEUROD1 protein is not expressed in the outer part of the avian and mammalian EGL. In the EGL of reptiles, avians, and mammals, ATOH1-positive GCPs (AT + GCPs) are highly proliferative and frequently undergo self-renewal. In reptiles and avians, GCPs cease proliferation after they lose ATOH1 expression and start expressing NEUROD1 instead. In the mammalian EGL, GCPs proliferate even after they start expressing NEUROD1 instead of ATOH1 (ND + GCPs). AT + GCPs are distributed in the upper part of the EGL and ND + GCPs in the middle part of the EGL in the mammalian cerebellum. Abbreviations: upper rhombic lip (uRL), external granule cell layer (EGL), internal granule cell layer (IGL), neural progenitor cells (NPCs), granule cells (GCs), ATOH1-positive granule cell progenitors (AT + GCPs), NEUROD1-positive granule cell progenitors (ND + GCPs).

The cerebellum is a brain region that rapidly increased in size and complexity during vertebrate evolution [8]. Interestingly, the developmental process is quite different among species, possibly leading to their different cerebellar structures (Figure 4, Table 1). In this section, we will compare the transit amplification systems of GC-lineage between species and discuss the evolutionary acquisition of the two-step amplification system for GCPs by mammals.

#### 3.2.1. Fish, Amphibians, Reptiles, and Birds

Cerebellar architecture can be observed in the lamprey [65]; however, it has a very small and simple plate-like structure. It has GC-like cells, but the presence of Purkinje cells is debatable. A rhombic lip-like structure, which is marked by the expression of *Pax6*, *Atoh1*, and *Wnt1* is also present [66]. The expression of *Pax6*, however, is weak and shifted more ventrally than in other vertebrates, possibly leading to the low degree of proliferation of its NSCs and smallness of its cerebellum-like structure. The cerebella of cartilaginous fish and teleosts are distinct but simply structured, with apparent GCs and Purkinje cells, as well as *Atoh1* and *Pax6* expression in the uRL of the cerebellar primordium [67,68]. In such fish, EGL-like structures are absent during cerebellar development; therefore, postmitotic GCs are generated directly from the uRL (Figure 4) [68]. An EGL-like structure has been observed in amphibians [69]. However, the cells in the amphibian EGL are not proliferative. They do not undergo self-renewal, unlike GCPs in the avian and mammal EGL (Figure 4), indicating no transit amplification systems in these animals. The transit amplification system has been reported in lizards and snakes [70]. In the EGL of lizards and snakes, GCPs undergo self-renewal to increase their progeny, suggesting that reptiles have acquired proliferative potential in the EGL (Figure 4). In the chick cerebellum, cell divisions occur frequently within the EGL, resulting in more complex lobule formation than that of reptiles (Figure 4) [71]. These studies have clarified that reptiles and birds possess a transit amplification system in which GCPs undergo further cell division after leaving the uRL. The increasing size of the cerebellum in reptiles and birds compared with fish and amphibians implies its positive correlation with the emergence of a transit amplification system.

#### 3.2.2. Rodents

In mammals, the size and morphological complexity of the cerebellum have increased considerably throughout the evolutionary process [72]. The biggest developmental difference between the chick and the mouse cerebellum is the expression of ATOH1 and NEUROD1 protein (Figure 4). In the chick cerebellum, in situ hybridization and *Neurod1*-promoter activity indicates that all GCPs in the EGL express *Atoh1*, while *Neurod1* is expressed only in postmitotic GCs [69,71]. However, in the mouse cerebellum, some GCPs express NEUROD1 protein instead of the ATOH1 protein, as revealed by tissue staining and scRNAseq [9]. An early study also supported this notion [73]. This comparison in expression patterns between the chick and mouse cerebellum suggests that ND + GCPs, a novel subtype of transit amplifying cells in the EGL, have been acquired in mammals, possibly leading to the observed cerebellar expansion. We have observed that ND + GCPs are uniformly distributed in the anterior-posterior and mediolateral axes during postnatal weeks 1–2 in the mouse cerebellum. However, detailed spatial and temporal distributions of ND + GCPs in the EGL during other developmental periods, such as the embryonic or perinatal, remain elusive. In the developing cerebral cortex, as described above, the balance between direct and indirect neurogenesis dynamically changes during development; direct neurogenesis predominates in the early stages and indirect neurogenesis in the late stages of development. Therefore, it is also possible that AT + GCPs generate ND + GCPs in the later stages of cerebellar development, while AT + GCPs directly generate GCs in the early stages.

#### 3.2.3. Humans

In apes, the cerebellum is reportedly larger and contains more cells than that of the other mammals, implying a link between cerebellar expansion and higher-order cognition [74]. A recent study has demonstrated that the relative surface area of the cerebellum is greater in humans (almost 80% of the surface area of the neocortex) than in macaques (only 33% of the surface area of the neocortex) [75]. Interestingly, crus I/II and lobules VIIb and VIIIa, which receive cortico-cerebellar input, are selectively enlarged in humans. The linkage between cerebellar size and cognitive functions is becoming evident in brain-imaging studies of patients with psychiatric disorders. Taken together, the size of the cerebellum is closely related to the acquisition and maintenance of higher-order cognitive functions, skillful movement, and language skills, all of which are remarkable features of human behavior. However, the molecular and cellular mechanisms that underlie human cerebellar enlargement remain unclear.

Early studies clearly described the development of human cerebellar GCs (aptly summarized by Marzban et al.) [76]; the formation of a rhombic lip is first observed at Carnegie stage (CS)16, which corresponds to the E14 rat cerebellum. Around CS21–CS23, the progenitor cells expand rostrally from the rhombic lip, which corresponds to the prospective EGL (the authors described it as an external germinal layer). Finally, the structure of the EGL is formed around the 10th gestational week (GW). In the upper layer of the EGL, the proliferation of GCPs is observed from the 18th GW, continuing past the postnatal period. During the fetal period, the cerebellum dramatically increases in size, as measured by using three-dimensional magnetic resonance imaging [77]. The proliferation rate of GCPs has been comprehensively studied with immunostaining for KI67. Accordingly, the KI67-labeled mitotic cells in the EGL were abundant during GW24–GW34, followed by a gradual decrease from GW36 to postnatal month 5 [78]. In postnatal months 8–11, the structure of the EGL is ambiguous, and the KI67-labeled cells disappear. Haldipular et al. also provided comprehensive anatomical data of the development of the human cerebellum after preterm birth [79]. Their observations suggest that a yearlong continuous proliferation of GCPs in the EGL from the fetal to the postnatal period results in the generation of numerous GCs in the human cerebellum, resulting in its expansion in size and complexity. At least two questions remain. (1) Do human cerebella undergo the same developmental process as rodents? (2) Do ND + GCPs exist in the human EGL, and, if so, are their mitotic activities or features comparable to those in mice? Importantly, we have confirmed that ND + GCPs are present in the developing human cerebellum by analyzing scRNAseq data (unpublished data), suggesting that the transit amplification system is a conserved hallmark of the mammalian cerebellum and may contribute to the expansion of the human cerebellum.

In recent anatomical and transcriptomic analyses of the human fetal cerebellum, it was suggested that the human uRL possesses structurally and functionally distinct features from those of rodents [80,81]. Moreover, Behesti et al. reported that the SOX2 protein, which is barely expressed in the mouse EGL, is clearly expressed in the human EGL [61]. Therefore, further study may reveal that humans have acquired a new transit amplification system to expand the number of GCs during their development. As developmental abnormalities in or injuries to the cerebellum are often related to defects in cognitive functions [82], further studies are required to advance our understanding of the pathology of such diseases and uncover the evolutionary mechanisms underlying cerebellar expansion.

## 4. Conclusions and Perspectives

In this review, we summarized the transit amplification of neural progenitors in certain species and brain regions, as well as evolutionary brain expansion. In particular, we focused on cerebellar transit amplification systems.

**Figure 5:**
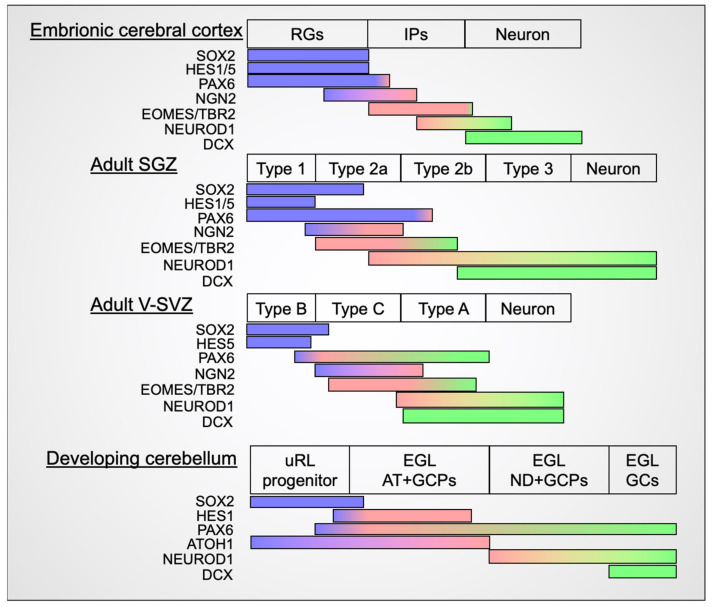
Similarities and differences in gene expression patterns during neurogenesis in different neurogenic regions of the mouse brain. Expression patterns of molecular markers during transit amplification of each brain region. SOX2 expression is a common feature of stem cells in every region (the cerebral cortex [85], the SGZ [86], the V-SVZ [35,86], and the cerebellum [61,87]), and DCX is a common feature of neurons (the cerebral cortex [88], the SGZ, the V-SVZ, and the cerebellum [88]). In addition, Hes family members are expressed in more undifferentiated progenitors (the cerebral cortex [89,90], the SGZ [91,92], the V-SVZ [93], and the cerebellum [62,94]). The cerebellum has interesting gene expression features compared to the other regions: (1) PAX6 expression is observed in the most immature progenitor and sustained after differentiation (the cerebral cortex [95], the SGZ [96,97], the V-SVZ [98,99], and the cerebellum [56,100]), whereas (2) expression of NGN2 (the cerebral cortex [101], the SGZ [99], and the V-SVZ [99]) and EOMES/TBR2 (the cerebral cortex [95], the SGZ [102], and the V-SVZ [99,103]) are not observed. NEUROD1 expression in mitotic cells is observed in the cerebrum, the SGZ, the V-SVZ, and the cerebellum [9,99,104,105,106]. Abbreviations: radial glia (RG), intermediate progenitor (IP), subgranular zone (SGZ), ventral subventricular zone (V-SVZ), upper rhombic lip (uRL), external granule cell layer (EGL), ATOH1 positive granule cell progenitors (AT + GCPs), NEUROD1 positive granule cell progenitors (ND + GCPs), granule cell (GC).

There are similarities and differences between transit amplification systems in the different brain regions in mice. Interestingly, the expression of many transcription factors is a common feature during transit amplification (Figure 5). *Hes1*/*5*, *Pax6*, and *Sox2* are expressed in stem cells and the most undifferentiated populations, whereas neurogenic genes such as *Neurod1*, *Tbr2*/*Eomes*, and *Ngn2* are often expressed in transit amplifying cells. The mechanism by which transit amplifying cells can undergo self-renewal despite expressing neurogenic genes that strongly promote cell-cycle exit and differentiation when overexpressed remains to be elucidated. One possibility is that these neurogenic genes work in a dose-dependent manner to suppress the cell cycle of transit amplifying cells. Although the dose-dependency of NEUROD1-mediated differentiation has been previously discussed, it remains unclear in the context of transit amplification [83]. Another possibility is the presence of transcriptional factors and/or signaling pathways that are expressed and/or activated in transit amplifying cells, suppressing neurogenic functions. We speculate that advances in scRNAseq will lead to the identification of such molecules and pathways by investigating the common features of transit amplifying cells among brain regions.

**Table 1:** Developmental processes vary among the species.

	uRL	EGL	ATOH1 Expression in the EGL	AT + GCPs	ND + GCPs
Fish [67,68]	Yes	No	No	No	No
Amphibian [69]	Yes	Yes (non-proliferative)	Yes	No	No
Reptile [70]	Yes	Yes	Yes	Yes	Unknown
Chick [71,84]	Yes	Yes	Yes	Yes	No
Mouse [9,61]	Yes	Yes	Yes	Yes	Yes
Human [61,80,81]	Yes	Yes	Yes	Yes	Yes

Differences between brain regions in transit amplification should also enhance our understanding of the molecular mechanisms regulating this process. For example, the timing and duration of transit amplification vary among the cerebral cortex, cerebellum, and SVZ/SGZ. In the mouse cerebral cortex, vast numbers of neurons are generated during the short embryonic period, while life-long neurogenesis occurs in the mouse SVZ/SGZ. The period of neurogenesis in the mouse cerebellum starts during the embryonic stage and lasts 2–3 weeks after birth. In addition, these periods vary across species. Investigation of these differences may advance our understanding of neurogenesis, as the progenitors have similar gene expression profiles and may use common principles in regulating the balance between proliferation and differentiation.

Although the transit amplification system efficiently amplifies neurons from a small number of stem/progenitor cells, the molecular machinery regulating this system remains elusive. The presence of transit amplification among widely different species and brain regions, as described in this review, hints at common molecular mechanisms. We hope that the unraveling of similarities and differences among species and brain regions will reveal the elegant molecular principles that control transit amplification in the brain. The evolutionary dynamics of cerebellar development may provide meaningful insights into solving these mysteries.
